# Opening the black box of under-health people: the case of Spain

**DOI:** 10.1186/s13561-016-0106-6

**Published:** 2016-06-24

**Authors:** Marta Pascual-Saez, David Cantarero-Prieto, Noelia González-Prieto

**Affiliations:** Department of Economics, University of Cantabria, Avda. de los Castros s/n, 39005 Santander, Spain

**Keywords:** Under-health, Matching techniques, EU-SILC, Earnings, I10, I14

## Abstract

The most famous modern definition of health was created during a Preamble to the Constitution of the World Health Organization in 1946: “Health is a state of complete physical, mental and social well-being and not merely the absence of disease or infirmity.” This definition has not been amended and, since then, many indicators have been proposed to measure health such as Self-Assessed Health (SAH) status. It provides an overall measure of a population’s health based on individuals’ personal perceptions of their own health.

In this paper, we focus our analysis on “under-health” as the fact of having a level that falls behind the health requirements necessary to perform what is considered an “expected life based on Self Assessed Health”. For Spain using the European Union Statistics on Income and Living Conditions (EU-SILC), we can confirm there exist under-healthy people by occupation, age group and sex. Additionally, under-healthy workers are most likely to be found among skilled agricultural, fishery workers and elementary occupations.

## Background

Empirical evidence in health economics and quality of life is largely based on different dimensions. In this sense, many studies have focused on the determinants of Self-Assessed Health (SAH) and its relationship with other relevant variables such as Gross Domestic Product (GDP), health care expenditure, income inequality, etc. As a result, over the past decades individuals’ health is specified as an individual characteristic function based on different socio-demographic inputs [1].

According to the literature, the key point is the effect of health status on labor productivity that is difficult to observe and measure. It requires finding an observable variable that is correlated with productivity. So, researchers have often used wages as a proxy of labor productivity. The validity of this idea is supported by standard economic theory that assumes that firms seek to maximize profit. This fact leads them to choose a level of labor hire where the cost of extra labor equals the increase in revenue related with the extra output from that labor. Therefore, profit-maximizing firms would be prepared to pay higher wages than other. The reason for it is to get more productive workers and health is related with.

As a determinant of human capital, health status makes an important contribution to individual’s productivity. The seminal paper of Grossman [2] on this topic attributed to health to be a durable capital stock. It produces an output of healthy time that is then allocated between leisure and work. So, poor (or under) health status restricts the amount of healthy time that may be allocated to generate more income.

Concomitant with it, one important difference between education and health status is that it is usually possible for employers to observe the education levels of employees (or potential employees) [3]. It is not surprising that employers can pay higher wages to more educated employees, if they consider that they are more productive. However, it is more difficult for employers to observe the health status of employees (or potential employees), and for employees to forecast their own health status.

In fact, labour status in terms of unemployment has a significant negative impact on both SAH and mental health [4]. This is to be expected and it has long been recognized in the literature that participation in work-related activities or physical activity yields positive results in terms of health outcomes and behaviors [5, 6].

Health measurement has been an issue of great debate within the economic literature. However, there remains much disagreement amongst health economists regarding the use of objective or subjective measures. Therefore, in this paper, we provide new empirical evidence about the impact of under-health (particularly under-SAH) on wages by using micro data for the Spanish adult population and the following methodology proposed by Duncan and Hoffman [7]. And to the best of our knowledge, it is among one of the first studies to analyse under-health in Spain and its implications. Indeed, we add to the debate by addressing the role of the socio-economic determinants through the Great Recession.

### Measuring under-health

Most of the empirical research is focused on indicators such as life expectancy, infant mortality, death rates, disability status or SAH. Thus, one of the most commonly used indicators of individuals’ health is SAH [1] which is classified into five categories. It reflects negative health rating (bad or very bad health) *versus* positive or neutral health ratings (very good, good or fair health).

Nevertheless, in health interview surveys, the SAH question is the key health variable available in most of the surveys and over the longest time period. Also, it is generally supplemented by other measurement tools, and its use is very common in other socio-economic surveys as, for instance, the European Statistics on Income and Living Conditions or the National Health Surveys.

In the past, researchers concerned with the measurement of health have dealt with the ordinal scale problem. Their results often differ depending on the choice of the cut points for healthy/non-healthy individuals. A variety of several approaches have been adopted in the health status scaling. In this context, under-health is a very new phenomenon and it is beginning to have negative effects on labour market output. Nevertheless, most of the previous research is devoted to over-education instead of health. That is, over-education occurs when investment in human capital exceeds that which is required for the job [8].

In the same way, we are going to apply this kind of methodology to health status. Therefore, under-health can be understood as the fact of having a level that falls behind the health requirements to perform a job. Thus, we define an “arbitrary” threshold around the mean (or the median) which takes the difference between the number of individuals above and below them. So, the main drawback is the loss of information as individuals are assigned as being above/below average regardless of how much better/worse they are. However, the advantage of this method is that it is more robust to outlier data than several other ones [9].

There are different methods to measure under-health and the mismatch of health similarly to over-education: the objective, subjective and statistical method [10]. Precisely, in this study we are going to focus our results on the method of interval. At this regard, the average person is defined as under-health if his/her health is less than one standard deviation bellow the mean of all individuals in their classification group: by occupation, group age or sex.

Beyond the mere effect of under-health, our main interest is to analyse the differential association between labour market and health status in Spain through the great recession period (2009–2012). A priori, we cannot expect an unambiguous effect but it depends on the dataset and methodology that we use.

### Methods

Empirically we have used information contained in the European Statistics on Income and Living Conditions (EU-SILC). The key advantage of this survey is that information is homogeneous among countries since the questionnaire is close across them. EU-SILC is an annual, EU-wide, survey which allows us to get information on the income and living conditions of different kinds of households and individuals in the European Union. It has been created to provide data to be used for the structural indicators of social cohesion. So, EU-SILC includes rich information about income, education, employment, health, etc. Moreover, it is designed to guarantee the comparability between the European Union countries.

EU-SILC includes a specific module on health, divided into three variables on health status and four variables on unmet needs for health care. The variables on health status represent the so called Minimum European Health Module (MEHM), and indicates three different concepts of health: Self-Assessed Health; Chronic Morbidity (people having a long-standing illness or health problem); Activity Limitation – disability (self-perceived long-standing limitations in usual activities due to health problems).

By EU countries, there also exist important differences. *Eurostat* (*2015)* [11] *publishes the report “Quality of life in Europe — facts and views”*. It presents different aspects of people’s well-being including health status and information is available on Eurostat’s website and within its online databases. From this report, it is important to point out that using EU-SILC (2012 and 2013) almost seven out of ten (67.7 %) EU residents reported being in good or very good health. Of these, 22.2 % actually reported being in very good health which is comparable to the shares of those who reported being in fair health (22.8 %). In 2013, Irish residents assessed their health status the least negatively (3.6 %), followed by the Maltese (3.9 %), Swedish (4.0 %), Dutch (5.4 %), Finnish (6.7 %) and Cypriot (6.9 %) residents. Additionally, Irish residents assessed their health most positively with 82.4 % of them declaring a good or very good health, followed by the Swedish (81.1 %), Cypriot (76.8 %), Dutch (75.4 %), and Greek (75.1 %) residents. By contrast, less than half of the Croatian (47.0 %), Lithuanian (46.3 %) and Latvian (45.4 %) residents stated to be in a good or very good health whilst between 16.5 % and 24.7 % of them perceived their health to be bad or very bad.

Thus, we are going to test the different hypothesis of the main determinants of under-health status in Spain. Self-Assessed Health is explained by a question on how a person perceives his/her health in general using one of the answer categories very good/ good/ fair/ bad/ very bad.

The employment status in current job is based on the International Standard Classification of Occupation – ISCO-88. The different categories are the following ones: 1) Legislators, seniors officials; 2) Professionals; 3) Technicians and associate professionals; 4) Clerks; 5) Service Workers and shop and market sales workers; 6) Skilled agricultural and fishery workers; 7) Craft and related trades workers; 8) Plant and machine operators and assemblers; 9) Elementary occupations. Also, we have classified individuals by age group and sex.

The data of this study are examined using recent matching techniques. These methods consider the causal effect in terms of potential outcomes or counterfactuals [12]. Let the variable *w* be a binary treatment indicator, where *w* = 1 denotes treatment and *w* = 0 otherwise. Following, Rosenbaum and Rubin [13] the average treatment effect on treated (*ATE*_1_) can be defined as

$$ AT{E}_1\equiv E\left({y}_1-{y}_0\left|\;w=1\right.\right) $$

where *ATE*_1_ is the average effect on participants in the program. In addition, we consider an individual *i*. He or she can receive the treatment (to be over-health) and his/her outcome is *y*_1_. If he/she do not receive the treatment (not to be over-health), then his/her outcome is *y*_0_. As well, (meta-)regression can be used to account for differences in measured covariates between subjects. However, this method may not fully adjust to evaluate non-experimental studies and alternative approaches based upon propensity scores have been proposed. These ones may provide more precise estimates of the treatment effect in observational studies in which confounding by indication may occur [13].

Once we have calculated the propensity score, we have several methods to make matching [14–16]. In particular, we are going to use nearest-neighbour matching, radius matching and stratification one. Firstly, nearest-neighbour matching will match the individuals whose propensity score has the smallest difference. Nearest-neighbour matching is defined by:

$$ C(i)=\underset{j}{ \min}\left\Vert {p}_i\right.-{p}_j\left\Vert, \right. $$

where *C*(*i*) is the set of control individuals matched to the treated individual *i* with an estimated value of the propensity of *p*_*i*_ and *p*_*j*_ is the propensity score of each individual of the control group.

Secondly, following radius matching, for the individual treated *i*, he or she will be matched with those individuals of the control group whose propensity scores are at a distance less than a given number,*r*:

$$ C(i)=\left\{{p}_j:\left\Vert {p}_i-\right.\left.{p}_j\right\Vert <r\right\}. $$

To test the sensitivity of our results we have considered different values for *r* (*r* = 0.1; *r* = 0.5; *r* = 0.01).

And, thirdly, we have applied stratification matching approach: The main idea of this method is to divide the common support region into intervals (or “blocks”) and then calculate the average treatment effect on treated for each interval. The overall *ATE*_1_*is* computed as weighted average of mean intervals effects, with weights being defined as the number of treated individuals in each interval. An estimator for the *ATE*_1_ using this method is given by:

$$ AT{E}_1={\displaystyle \sum \left(AT{E}_{1K}x{N}_{T\in K}^{*}\right)\frac{1}{N_T^{*}},} $$

where *ATE*_1*K*_ is the *ATE*_1_ for each interval *K and*${N}_T^{*}$ denote the number of treated individuals falling with the common support region and in interval *K*.

For this analysis, we are going to take into account the following characteristics according to the different categories based on the EU-SILC definitions commonly used: income, gender, age (in years), marital status, education level, sector of employment and health status.

## Results

As we mentioned before, the variable we employ as proxy of individual’s health status is the SAH that each individual reports of its own health status and the possible responses are ordered qualitatively. Therefore, SAH variable is a subjective response to the question “How is your heath in general?” and it takes the values “5” (very good), “4” (good), “3” (fair), “2” (bad) and “1” (very bad).

Let us begin by looking at some current health level data. Tables 1 and 2 provide the proportion of individuals defined as over-health, adequate-health and under-health within each of the broad categories. Thus, under-health workers are most likely to be found among “Skilled agricultural and fishery workers” and “Elementary occupations”, around 50 and 40 % respectively. There are also great differences by sex and group age. At this regard, under-Health individuals are mainly male (25.28 % in 2012 and 24.84 % in 2011) and between 39 and 50 years old (15.83 % in 2012 and 16.13 % in 2011). Also, it is important to highlight that the results obtained using the average criteria do not differ very much from those obtained with the mode one.

**Table 1:** Evolution on under-health and over-health in Spain 2009-2012 by occupation: Method of interval

	% under-health				% over-health				% adequate-health			
	2009	2010	2011	2012	2009	2010	2011	2012	2009	2010	2011	2012
1.- Legislators, seniors officials	26,43	25,96	22,19	19,26	13,95	16,44	16,56	18,18	59,62	57,60	61,26	62,55
2.- Professionals	12,74	12,40	11,71	11,57	21,31	22,52	26,45	28,00	65,95	65,08	61,84	60,43
3.- Technicians and associate professionals	17,00	15,02	17,52	18,81	21,44	22,45	23,47	23,91	61,56	62,53	59,02	57,28
4.- Clerks	18,83	19,18	17,53	15,88	17,64	18,16	23,66	20,57	63,53	62,66	58,81	63,55
5.- Service workers and shop and market sales workers	27,24	26,19	22,67	23,15	14,91	16,80	20,81	21,84	57,86	57,02	56,52	55,02
6.- Skilled agricultural and fishery workers	53,04	53,44	48,70	49,23	6,08	6,43	10,54	6,40	40,88	40,13	40,76	44,37
7.- Craft and related trades workers	33,97	34,40	31,60	32,82	11,69	12,23	15,34	14,80	54,34	53,37	53,06	52,38
8.- Plant and machine operators and assemblers	32,62	32,22	28,68	26,99	11,33	13,30	16,16	16,82	56,05	54,47	55,16	56,18
9.- Elementary occupations	41,98	41,90	34,76	38,75	10,52	11,31	14,38	15,23	47,50	46,79	50,87	46,02
Total	15,17	14,13	11,00	11,22	18,02	19,18	23,69	24,00	66,82	66,69	65,31	64,78

Source: Authors’ elaboration on EU-SILC

**Table 2:** Evolution on under-health and over-health in Spain 2009-2012 by group age and sex: Method of interval

	% under-health				% over-health				% adequate-health			
By age	2009	2010	2011	2012	2009	2010	2011	2012	2009	2010	2011	2012
25 < Age < 40	13,57	12,71	9,91	9,52	22,48	25,16	32,27	33,78	63,95	62,13	57,82	56,70
39 < Age < 50	21,99	20,70	16,13	15,83	13,58	15,30	19,12	20,16	64,43	63,99	64,76	64,01
49 < Age < 65	10,60	9,86	8,62	8,72	8,43	8,62	11,27	10,99	80,98	81,52	80,11	80,29
By Sex	2009	2010	2011	2012	2009	2010	2011	2012	2009	2010	2011	2012
Male	9,06	8,87	24,84	25,28	16,14	17,23	21,02	21,75	74,80	73,90	54,15	52,98
Female	11,80	11,38	10,25	10,68	13,42	14,78	18,95	19,34	74,78	73,84	70,80	69,98

Source: Authors’ elaboration on EU-SILC

In Spain, as we expected, there exists significant under-SAH levels by occupation, age group and sex. In order to test this hypothesis we are going to consider different socio-demographic variables which could explain these differences.

Our estimation results using STATA 12.0 are reported in Table 3 (“nearest neighbour”, “radius matching” and “stratification” method). Overall, we can check that under-health has in all the cases a negative and significant impact on earnings. As we have previously observed, these findings remain even if we divide our sample into two subsamples (males and females). In addition, for the individuals of our sample, the average effect of being under-health in 2012 is a decrease of the annual earnings (wages) by 2067.49 euros for males and 1017.78 euros for females (stratification matching method). These results suggest that being under-health increases the incidence of individual’s status on wages. Furthermore, the estimates are quite robust to the matching method.

**Table 3:** *ATE*_*1*_ of under-health on wages in Spain 2012

	Method of interval								
	Total			Male			Female		
	*ATE*_*1*_	Std. Err.	t	*ATE*_*1*_	Std. Err.	t	*ATE*_*1*_	Std. Err.	t
Nearest neighbour	−1555.231	160.288	−9.703	−2096.126	262.021	−8.000	−963.914	184.987	−5.211
Radius matching (0.1)	−3430.540	129.377	−26.516	−5164.229	199.292	−25.913	−1922.907	162.608	−11.825
Radius matching (0.05)	−3420.403	129.727	−26.366	−5299.931	199.234	−26.601	−1776.941	163.845	−10.845
Radius matching (0.01)	−3259.498	130.193	−25.036	−5603.878	200.019	−28.017	−1078.173	171.353	−6.292
Stratification	−1578.426	112.184	−14.070	−2067.496	168.905	−12.241	−1017.777	140.366	−7.251

Source: Authors’ elaboration on EU-SILC

## Discussion

In this paper, using the European Statistics on Income and Living Conditions we analyze the problem of over-health and under-health and their effects on earnings in Spain. It is a useful exercise to put the results of this work into context in terms of magnitude. So, in our study about 12 % of individuals were found to be “underhealth people”. Obviously, this percentage varies among occupation, age group and sex. Besides, the above-mentioned analysis is carried out based on propensity score matching techniques.

Additionally, our estimation results are based in several matching techniques (“nearest neighbour”, “radius matching” and “stratification” method”). At this regard, under-health workers are most likely to be found among these groups: skilled agricultural, fishery workers and elementary occupations. It means that under-health has a significant negative impact on earnings. Thus, those individuals, who declare worse SAH, receive fewer rewards. Moreover, this findings remains even if we divide our sample into males and females. The previous output implies that for the individuals of our sample, the average effect of being under-health in 2012 is a decrease of the annual earnings (wages) by 2067.49 euros for males and 1017.78 euros for females. These results suggest that estimates are quite robust to the different matching method that we use and it can be spread for further research analyzing under health evolution in the European Union countries. Despite concerns about subjective measures of health as self-assessed status, international studies have found it has strong predictive power for mortality.

It is also important to highlight the research limitations of the study. The EU-SILC allows the incorporation of individual-specific characteristics. So, these data are self-reported and imply that the empirical results are tentative. It is similar to the relationship between happiness and health which is found positively correlated by other researchers [17] or the heterogeneity in health among elderly people [18].

Although these studies are based on subjective data their results are in line of our findings. So, they demonstrate that being married, educated are positively associated with being happy and being healthy. Conversely, individual unemployment is negatively associated with happiness and health. But instead of dampening researchers’ spirits, these notes should serve to focus further research into an issue of vital importance to Spain’s citizens, under-health situations and growing inequalities in last decades. On the other hand

## Conclusions

In this paper, we concentrate our analysis on under-health as the fact of having a level that falls behind the health requirements necessary to perform what is considered an “expected life based on Self Assessed Health”. For Spain, using the EU-SILC, there exists under-SAH by occupation, age group and sex. As a result, it was found that about 25.28 % of males were considered “under-health people” in 2012. This data reflects an increase in “underhealth people” in last years because it was only 9.06 % in 2009. Probably, the Great Recession and public health cuts in Spain based on austerity measures are the reason for these phenomenon. The main findings of this paper add more empirical evidence supporting the effect of health status on individuals’ earnings.

The main findings of this paper add more empirical evidence supporting the effect of health status on individuals’ earnings. Despite concerns about subjective measures of health as self-assessed status, international studies have found it has strong predictive power for mortality.

These results also yield strong policy implications for the health care sector. So, one of the main objectives for policy makers should be clearly directed to improve population health, especially at lower paid people that have been more damaged by economic crisis. Our results provide new empirical evidence on the relevance of incorporating the health dimension to identify different employment profiles.
